# Mistranslation and its control by tRNA synthetases

**DOI:** 10.1098/rstb.2011.0158

**Published:** 2011-10-27

**Authors:** Paul Schimmel

**Affiliations:** Departments of Molecular Biology and Chemistry, The Skaggs Institute for Chemical Biology, The Scripps Research Institute, La Jolla, CA 92037, USA

**Keywords:** genetic code, editing, serine-for-alanine mistranslation

## Abstract

Aminoacyl tRNA synthetases are ancient proteins that interpret the genetic material in all life forms. They are thought to have appeared during the transition from the RNA world to the theatre of proteins. During translation, they establish the rules of the genetic code, whereby each amino acid is attached to a tRNA that is cognate to the amino acid. Mistranslation occurs when an amino acid is attached to the wrong tRNA and subsequently is misplaced in a nascent protein. Mistranslation can be toxic to bacteria and mammalian cells, and can lead to heritable mutations. The great challenge for nature appears to be serine-for-alanine mistranslation, where even small amounts of this mistranslation cause severe neuropathologies in the mouse. To minimize serine-for-alanine mistranslation, powerful selective pressures developed to prevent mistranslation through a special editing activity imbedded within alanyl-tRNA synthetases (AlaRSs). However, serine-for-alanine mistranslation is so challenging that a separate, genome-encoded fragment of the editing domain of AlaRS is distributed throughout the Tree of Life to redundantly prevent serine-to-alanine mistranslation. Detailed X-ray structural and functional analysis shed light on why serine-for-alanine mistranslation is a universal problem, and on the selective pressures that engendered the appearance of AlaXps at the base of the Tree of Life.

## 1. Introduction

**Figure 1.**
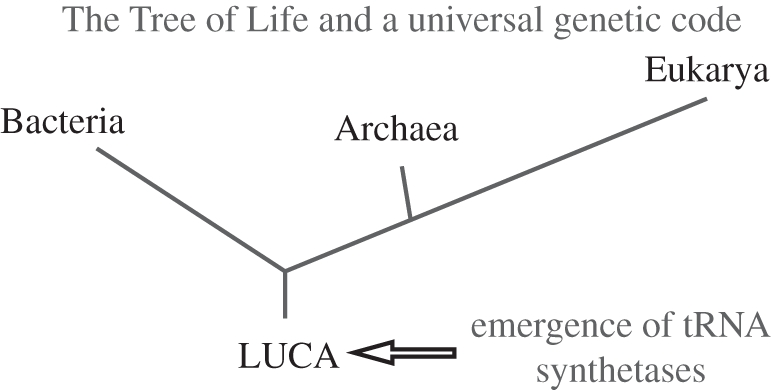
The universal Tree of Life with emergence of tRNA synthetases at the base of the Tree. LUCA, last universal common ancestor.

The genetic code was perhaps the greatest discovery of the twentieth century. Most striking was the concept that all forms of life—simple micro-organisms, pterodactyls, great mammoths, dolphins, finches and humans—were different manifestations of the same universal code that was imbedded in the genetic material. The code is a simple algorithm that relates each of 20 amino acids to specific nucleotide triplets. These relationships are established by aminoacyl tRNA synthetases—enzymes that catalyse the aminoacylation reactions that attach each amino acid to its cognate tRNA that, in turn, harbours the anticodon triplets of the code [1,2]. The aminoacylation reaction is the first step of protein synthesis and results in the production of Ala-tRNA^Ala^, Ile-tRNA^Ile^, Gln-tRNA^Gln^ … . As essential enzymes needed to interpret the genetic material, the tRNA synthetases are thought to have arisen during the transition from the RNA world to the theatre of proteins. Thus, they appeared at the base of the Tree of Life before it expanded to three great kingdoms (figure 1).

Recent work has revealed the causal connection of pathologies and even heritable genetic mutations to mistranslation of genetic information by tRNA synthetases. The work also has demonstrated the powerful mechanisms that control mistranslation, using specialized activities of tRNA synthetases as well as of a few novel genome-encoded accessory proteins specifically designed to provide a redundant mechanism to further limit mistranslation. These mechanisms to control mistranslation can be traced back to the base of the Tree of Life, thus suggesting that extant organisms could not exist without them. Interestingly, the mistranslation of serine for alanine, which creates Ser-tRNA^Ala^ and can result in the insertion of serine at places in proteins that are reserved for alanine, is an ancient problem and paradox that affects organisms throughout evolution. Ser-to-Ala mistranslation has been connected to disease in mammals. Phylogenetic, structural and functional analysis suggests that the problem of serine-for-alanine mistranslation may have been the most difficult for living organisms to solve. Summarized below is an overview of this problem and the unconventional solution to overcome it.

## 2. The phenomenon of statistical proteins

An early genetic code may have treated similar amino acids as equivalent. Examples would be isoleucine, leucine and valine being considered as interchangeable, and likewise for the aromatic amino acids—tyrosine, phenylalanine and tryptophan [3]. In this scenario, a polypeptide encoded by the genome would be a statistical entity, that is, a collection of similar sequences in admixture. Statistical proteins could have some advantages in an early environment for the evolution of life. For example, in contrast to a modern enzyme with exact specificity, because of the subtle variations in the substrate recognition site created by the statistical ensemble of sequences, an enzymatic activity associated with a statistical protein could act on a variety of closely similar substrates. In addition, statistical proteins can explore a larger amount of ‘sequence space’ to find the optimal sequence for a defined function. However, while statistical proteins occur naturally in a limited way in, for example, *Candida albicans* [4], or by engineering of *E. coli*, they are special cases [5]. Modern organisms, from bacteria to mammals, are adversely sensitive to statistical polypeptides [6–9]. This sensitivity is manifested in two ways. First, the statistical proteins themselves are toxic and can, in mammalian cells, lead to the unfolded protein response and apoptosis [9,10]. Second, as shown in ageing bacteria, mistranslation is mutagenic, because of the DNA damage over many generations and the resulting errors of replication that come from the error-prone DNA repair system [11].

## 3. Control of mistranslation by editing activities

Some aminoacyl tRNA synthetases are inherently unable to discriminate against amino acids that are closely similar to the cognate one [12–14]. An example is isoleucyl-tRNA synthetase (IleRS), which misactivates valine at a frequency of roughly 1/200 [15]. Isoleucine can only be recognized by hydrophobic interactions and valine, which lacks one methylene group compared with Ile, can fit into the same binding pocket on the enzyme. Likewise, valyl-tRNA synthetase (ValRS) misactivates threonine, which is virtually isosteric with Val. These examples make clear that misactivation is generally associated with amino acids that are similar to, but smaller than, the cognate amino acid. In these examples, the amino acid that is misattached to tRNA is removed by a separate hydrolytic editing activity, which can act in *cis* (that is, before the mischarged tRNA is released from the synthetase) or in *trans* (by rebinding of the released mischarged tRNA and subsequent clearance of the non-cognate amino acid) [16–21].
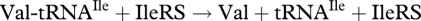
and
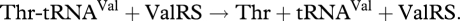


Paradoxically, alanyl-tRNA synthetase (AlaRS) misactivates *both* glycine (smaller than alanine) and serine (larger than alanine) [22]. While the misactivation of Gly by AlaRS is not surprising, misactivation of serine cannot be understood as the accommodation of a smaller amino acid in the amino acid binding pocket. In both instances, the same active site in AlaRS removes the mischarged amino acid from tRNA^Ala^.
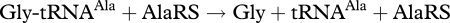
and
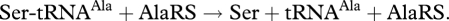


The ‘serine paradox’ is deeply rooted in evolution and reflects some of the limitations in the historical design of the protein synthesis apparatus [23]. At the same time, it resulted in an unconventional solution that is also deeply rooted.

## 4. Serine-to-alanine mistranslation in the mouse

The spontaneous *sti* mutation in the mouse gives rise to severe neurological disease that is manifested by ataxia and progressive degeneration of Purkinje cells in the cerebellum [10]. This ‘sticky mouse’ mutation (named because of the sticky characteristics of the fur) renders the animal sensitive to serine-to-alanine mistranslation. This sensitivity is clearly seen by the toxicity of serine when added to primary cells cultured from the *sti* mouse. In contrast, the same cells showed little sensitivity to added glycine.

**Figure 2.**
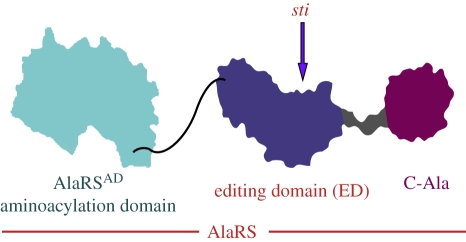
Domains of AlaRS arranged in a linear fashion along the sequence. This arrangement of domains is conserved in all three kingdoms of the Tree of Life. The location of *sti* mutation associated with neurological degeneration in the mouse is noted.

AlaRS is the most conserved tRNA synthetase through evolution [10]. The protein has three major domains that are arranged in a linear fashion along the polypeptide sequence. Starting from the N-terminus, there is a domain for aminoacylation (AD), another for editing (ED), and a third at the C-terminus that is designated as C-Ala (figure 2). The *sti* mutation is a single Ala → Glu substitution in the ED [10]. The mutation is mild—for example, the activity for deacylation of Ser-tRNA^Ala^ is reduced only twofold. Although the mutation is recessive, it is likely that a stronger mutation would be dominant and lethal. By analogy, in mammalian cells that were induced to express an editing-defective ValRS transgene, small amounts of Val-tRNA^Thr^ were accumulated and resulted in a dominant phenotype of cell pathology and apoptosis [9].

## 5. Understanding the deeply rooted serine paradox

The aminoacylation reaction proceeds through an amino acid activation step in which the amino acid is condensed with ATP to form the tightly bound aminoacyl adenylate (AA-AMP). The bound adenylate is then reacted with the 3′-end of the cognate tRNA to give AA-tRNA. To understand the root of the serine paradox, nine co-crystal structures were solved, with AlaRS bound to alanine, serine, an ATP analogue and stable aminoacyl adenylate analogues 5′-O-(N-(l-alanyl)-sulphamoyl adenosine) (Ala-SA) and Ser-SA. Collectively, these structures provided a snapshot of the amino acid activation step, and of the reason behind the activation of serine [23].

**Figure 3.**
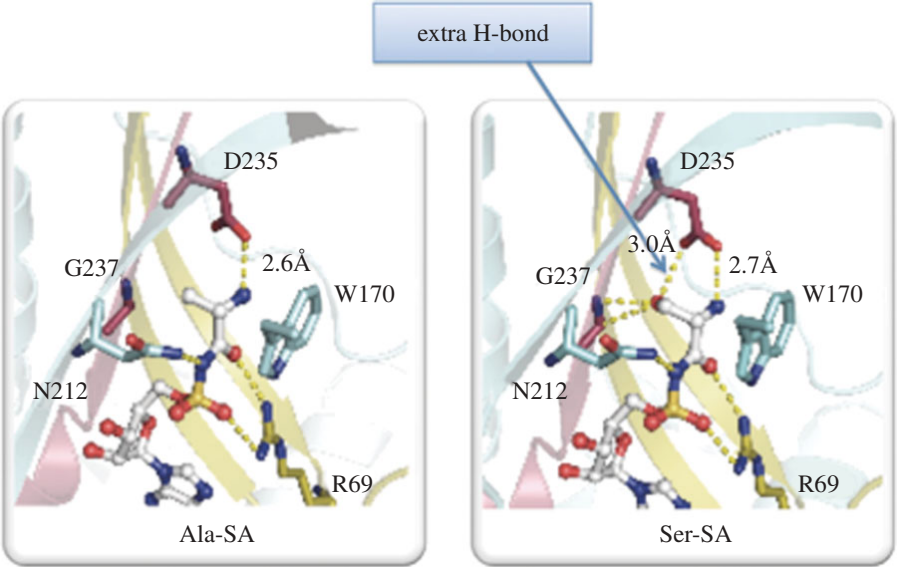
Active site of AlaRS with bound adenylate analogues (Ala-SA and Ser-SA). A universally conserved Asp carboxylate pins down the α-amino group of the bound adenylate. This same carboxylate makes a serendipitous H-bond with the sidechain -OH of bound serine.

The α-amino group of the bound amino acid is pinned down by the side chain carboxyl of a universally conserved active site aspartate. At the same time, this design creates a serendipitous interaction with the side chain -OH of serine, thereby enabling it to be bound and condensed with ATP (figure 3). This architecture dates back 3 billion years and apparently reflects the inability of nature to find an alternative solution that avoids the serendipitous interaction with the Ser -OH [23].

To further investigate the constraints that resulted in this design, we used site-directed mutagenesis to change the conserved Asp to Glu, Asn and Gln. None of the three mutations improved the wild-type enzyme's discrimination (about 1/300) between Ala and Ser, and at the same time made the enzyme much less efficient with alanine. For example, the Asp → Asn substitution reduced the discrimination between Ala and Ser from about 1/300 to 1/5. The same mutation also reduced the discrimination between Ala and Gly (from 1/170 to 1/3). In another vein, shrinking the binding pocket to squeeze out serine was also attempted. This shrinkage was accomplished by replacement of a Gly in the binding cavity with the more bulky Ala. This substitution did not greatly affect the *K*_m_ for Ser, but raised the *K*_m_ for Ala by 1500-fold. Co-crystal structures of the mutant enzyme with each of the bound amino acids clearly showed the crowding of alanine by the shrunken pocket of the Gly → Ala mutant protein, whereas serine still retained the serendipitous interaction with its bound -OH [23].

For other tRNA synthetases, the mechanisms for pinning down the α-amino group of the bound amino acid are idiosyncratic. Interestingly, for both SerRS and ThrRS, the same group (glutamate or zinc) that binds the α-amino group is also used to bind the side chain γ-OH [23–25]. ThrRS is another tRNA synthetase known to misactivate a larger amino acid—hydroxynorvaline. However, hydroxynorvaline does not occur naturally and, therefore, presents no dilemma for the architectural design of ThrRS [25].

## 6. Alaxp as a solution to the serine paradox

**Figure 4.**
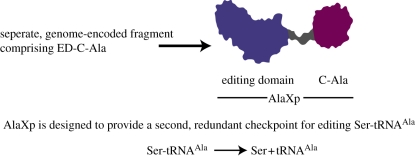
Schematic of the free-standing AlaXp editing domains (EDs) that is homologous to the ED and C-Ala portions of AlaRS (figure 2).

AlaRS is apparently unique in having an insurmountable challenge to prevent the misactivation of a larger amino acid (serine). Perhaps for this reason, free-standing AlaXp's appeared in order to provide a redundant mechanism for clearance of Ser-tRNA^Ala^ [7,26–30]. (Interestingly, many AlaXps are specific to Ser-tRNA^Ala^ and have little or no activity on Gly-tRNA^Ala^ [31].) The AlaXps encode the editing domain (ED) of AlaRS and arose contemporaneously with the first AlaRSs. In most eukaryotes and many archaea and bacteria, the ED is fused to a homologue of the C-Ala domain, thus making these AlaXps similar to the entire C-terminal half of AlaRS (figure 4). The only other free-standing EDs annotated so far are ones corresponding to the ED of ThrRS and the YbaK protein that clears Cys-tRNA^Pro^ [32,33]. Unlike the AlaXps that are distributed through all three kingdoms of the Tree of Life, the free-standing ED of ThrRS is limited to certain crenarchaeal species [34], and the Ybak ED is also limited in its distribution.

## 7. Distinct aminoacylation and editing domains recognize the same base pair

**Figure 5.**
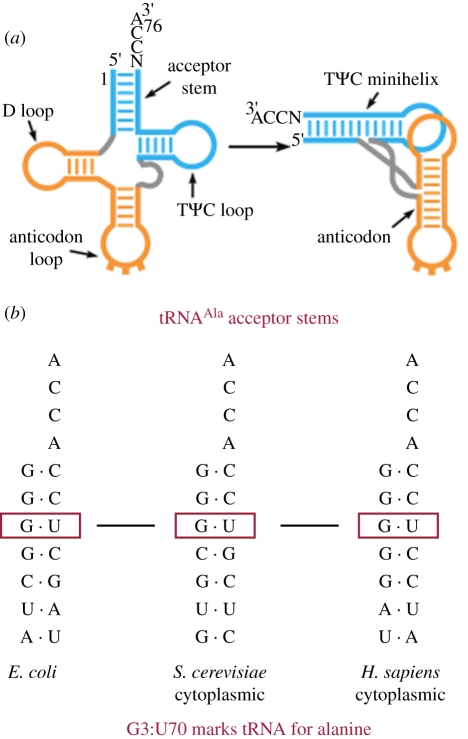
(*a*) Schematic of the tRNA cloverleaf secondary structure (left) and its folding into an L-shaped three-dimensional structure (right). Common landmarks on the tRNA are noted. (*b*) Acceptor stems of bacterial, yeast and human tRNAs specific for alanine and the conserved G:U base pair that marks the tRNA for aminoacylation with alanine.

Transfer RNAs are typically 76 nucleotides that fold into a cloverleaf secondary structure that, in turn, folds into an L-shaped tertiary structure (figure 5*a*) [35]. The amino acid attachment site is a terminal adenosine at the 3′-end of one arm (the ‘acceptor arm’) of the L, which ends in the sequence NCCA_3′OH_. The anticodon is at the end of the other arm of the L, separated by 76 Å from the amino acid acceptor site. A single G:U base pair, located proximal to the amino acid attachment site, marks a tRNA for aminoacylation with alanine. This G:U base pair is universally distributed in tRNA^Ala^s and, from bacteria to humans, has been shown to be the major determinant for aminoacylation with alanine (figure 5*b*) [36–40]. The recognition of this base pair by AlaRSs is so robust that the transfer of G:U into non-alanine tRNAs converts them into alanine acceptors. In addition, oligonucleotide substrates that contain only a few base pairs from the end of the acceptor arm are robust substrates, provided they encode G:U [41]. Because the G:U base pair is distinct from and distal to the anticodon triplet of the code, the relationship between alanine and the nucleotide triplet that corresponds to alanine is indirect. This observation has been expanded to other examples where nucleotide determinants for specific aminoacylation are proximal to the amino acid acceptor site [28,30,42]. Relating nucleotide determinants in the acceptor arms of tRNAs to specific amino acids has been proposed as a ‘second genetic code’.

The deacylation of Gly- or Ser-tRNA^Ala^ by AlaRS is also sensitive to the presence of G:U. Remarkably, there are two acceptor-arm binding sites on AlaRS that recognize G:U. These sites are entirely distinct and have no sequence or structural similarity. One is located in the AD (as part of a 10-helix bundle) and the other in the ED (associated with a β-hairpin) [28,30,42]. A recombinant fragment consisting of just the AD aminoacylates tRNA^Ala^ (as well as oligonucleotide substrates encoding a small portion of the acceptor arm) with the same dependence on G:U as the native enzyme. A separate recombinant fragment, consisting of ED–C-Ala, deacylates Gly- or Ser-tRNA^Ala^ in a G:U-sensitive reaction. Similarly, AlaXp, which is homologous to ED–C-Ala, deacylates Ser-tRNA^Ala^ with G:U-sensitivity.

In contrast to the aminoacylation reaction, where G:U-encoding RNA oligonucleotide substrates are active for aminoacylation, the same constructs (aminoacylated with Ser) are not active in the editing reaction. In the editing reaction, the full tRNA is required [43]. This requirement reflects the function of the elbow, or corner, of the L-shaped tRNA structure in coordinating the AD with the ED.

## 8. The structure and role of the C-Ala domain

The C-Ala domain is joined to the ED of all AlaRSs and in many of the AlaXps. This strong conversation of C-Ala motivated us to determine its three-dimensional structure [29]. The 1.85 Å structure of the 110-amino acid fragment revealed a central six-stranded β-sheet with flanking helices, arranged into a globular shape. The C-terminal 70 amino acids are closely similar in structure to the DHHA1 domain of the RecJ exonuclease that binds to single-stranded DNA. This observation motivated further studies to investigate the interaction of C-Ala with tRNA^Ala^. In these investigations, RNA footprint analysis showed that C-Ala specifically protected the D-loop portion of tRNA^Ala^, and thus supported the idea that it is designed to recognize the L-shape. Interestingly, the *K*_d_ for binding of C-Ala to tRNA^Ala^ (about 1 µM) is approximately the same as that for binding of ED–C-Ala (about 2 µM). These results show that C-Ala has an important role in providing stability to the synthetase–tRNA complex.

**Figure 6.**
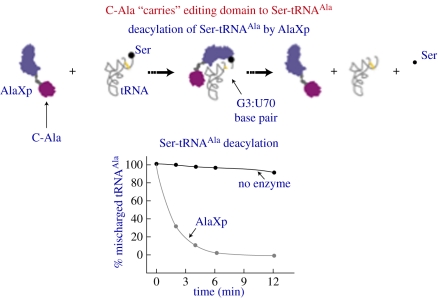
Schematic of how the C-Ala domain of AlaXp carries the ED to Ser-tRNA^Ala^. The C-domain binds to the outside corner of the L-shaped tRNA.

Strikingly, while ED–C-Ala and C-Ala have approximately the same affinity for tRNA^Ala^, the removal of C-Ala from ED reduces the editing activity approximately 1000-fold compared with the activity of AlaRS. And fusing C-Ala and its linker (that joins it to ED) from human AlaRS to *E. coli* ED gives almost full activity for deacylation of Ser-tRNA^Ala^. This observation suggested a strong conservation of function for C-Ala and its linker. This function is to ‘carry’ the ED to Ser-tRNA^Ala^ [29]. An example using AlaXp is shown in figure 6.

**Figure 7.**
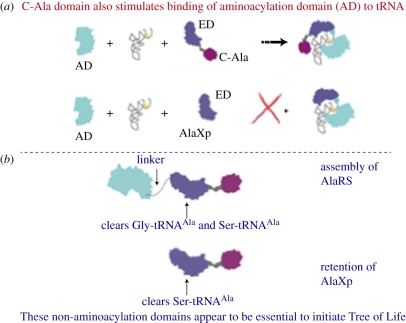
(*a*) Schematic showing that C-Ala brings together aminoacylation and editing functions on the same tRNA. (*b*) The editing function of AlaRS is not sufficient by itself to maintain cell homeostasis and for that reason free-standing AlaXp's were retained to provide redundancy for clearing Ser-tRNA^Ala^.

In addition, in electrophoresis gel shift experiments, ED–C-Ala dramatically stimulated the binding of AD to tRNA, and thereby sharply raised the aminoacylation activity encoded by AD. With the addition of ED alone, binding of AD to AlaRS was significantly reduced and there was no effect on aminoacylation. These results showed that C-Ala brings together the aminoacylation and editing functions on one tRNA (figure 7) [29]. When AD and ED are brought together on the same tRNA, computer models—based on three-dimensional structural determinations of AD, ED and C-Ala—suggested that AD and ED approach the acceptor arm of tRNA^Ala^ from opposite sides, where one domain interacts with the major groove side of the G:U pair, and the other with the minor groove side.

## 9. Assembly of alars in evolution and conclusions

The aforementioned experiments support the possibility that AlaRS started as an AD that non-covalently associated with ED–C-Ala. The C-Ala moiety of ED–C-Ala was specifically designed to bring together the aminoacylation and editing functions on one tRNA. Over time, the three domains were joined together in one protein. Consistent with this interpretation, AlaXp can be traced back to the base of the Tree of Life, showing that it appeared contemporaneously with early AlaRSs [29]. However, because of the extreme sensitivity of cell homeostasis to mistranslation of serine-for-alanine, AlaXp was retained in all three of the kingdoms of life. The retention of AlaXp gave an additional checkpoint for clearance of Ser-tRNA^Ala^. Thus, any Ser-tRNA^Ala^ that escaped the editing activity of AlaRS was still subjected to editing by AlaXp.

In the *sti* mouse, a twofold reduction in the editing activity of AlaRS resulted in a severe neurological pathology that comes from serine-for-alanine mistranslation. Because the *sti* mouse encodes wild-type AlaXp, this severe phenotype emphasizes the need for functional redundancy of the activity for removing Ser-tRNA^Ala^. Indeed, in a converse experiment that further supported the need for functional redundancy, RNAi suppression of AlaXp levels in mammalian cells triggered the unfolded protein response that is characteristic of cells approaching apoptosis [44].
